# *Cistanche tubulosa*-*Ginkgo biloba* combination enhances memory via cortico-cerebellar reorganization: a randomized controlled trial

**DOI:** 10.3389/fphar.2026.1654013

**Published:** 2026-03-02

**Authors:** Yuhong Gao, Binrui Yang, Wei Wang, Yao Meng, Rong Luo, Liang Chen, Jun Du

**Affiliations:** 1 Department of Neurology, Second Medical Center, PLA General Hospital, Beijing, China; 2 Amway (Shanghai) Innovation & Science Co., Ltd., Shanghai, China; 3 Amway (China) Co., Ltd., Guangzhou, China; 4 Department of Diving and Hyperbaric Medicine, Naval Special Medical Center, Naval Medical University, Shanghai, China

**Keywords:** *Cistanche tubulosa*, cognitive aging, cortico-cerebellar reorganization, fMRI, *Ginkgo biloba*, gray matter volume, neuroplasticity

## Abstract

**Background:**

Age-related memory decline has emerged as a critical global public health challenge, yet few ethnopharmacological interventions have demonstrated robust clinical efficacy in improving memory and cognitive function.

**Objectives:**

This randomized, double-blind, placebo-controlled trial aimed to elucidate the neurocognitive-enhancing mechanisms of a botanical product containing *Cistanche tubulosa* [Schenk] Wight and *Ginkgo biloba* L. (CG) extracts.

**Methods:**

117 healthy participants (aged 30–65 years) were randomized to receive either CG tablets or placebo for 30 days. Clinical memory assessments and MRI scans were conducted at baseline and post-intervention. Gray matter volume (GMV), functional MRI (fMRI), and functional connectivity (FC) were analyzed using SPM12.

**Results:**

CG tablets significantly improved scores in across multiple dimensions, including directional memory, associative learning, graphic free recall, portrait memory, total memory, and Memory Quotient (all *p* < 0.01). Subgroup analyses by age (<50 vs. ≥50) and gender confirmed the consistency of these effects. After intervention, slight reductions in GMV were observed in precentral gyrus and supplementary motor area. Notably, greater improvement in clinical memory scores was associated with lower post-intervention GMV in these regions. Concurrently, fMRI revealed increased activity in the cerebellar culmen, which positively correlated with improvements in directional memory (p = 0.031) and total memory score (p = 0.007). The associations between memory improvement, localized GMV reduction, and increased cerebellar activity suggests that CG may optimize neural efficiency or drive functional network reorganization.

**Conclusion:**

These findings indicated that CG supplementation enhances memory function in healthy adults. These cognitive gains are linked to specific structural and functional neural changes, particularly in motor-related areas and the cerebellum, reflecting improved neural processing. While these findings highlight CG’s short-term benefits, further research is warranted to explore its long-term efficacy and broader clinical utility.

This study was registered at the Chinese Clinical Trials Registry (https://www.chictr.org.cn/): ChiCTR2400084102.

**Clinical Trial Registration:**

https://www.chictr.org.cn/hvshowproject.html?id=251905&v=1.0, identifier ChiCTR2400084102.

## 1 Introduction

Life expectancy has increased markedly since the last century, leading to a rapidly expanding elderly population globally. This demographic shift underscores the urgent need to address age-related health challenges, particularly cognitive decline, as a critical public health priority (Aburto et al., 2020). Neurostructural alterations, including reductions in brain volume, hippocampal atrophy, and cortical thinning, often emerge as early as the fourth decade of life, with memory impairment being a primary manifestation (Gaspar-Silva et al., 2023). Given memory’s fundamental role in learning, decision-making, and overall quality of life (Maldonado Briegas et al., 2020), identifying effective interventions to preserve cognitive function is essential.

The growing demand for improving memory and cognitive function has catalyzed numerous randomized controlled trials (RCTs). For instance, a double-blind RCT confirmed that supplementation with specific probiotic strains may enhance cognitive function (Kim et al., 2021). Similarly, a meta-analysis of 23 RCTs found that creatine supplementation improved memory in healthy individuals, particularly in older adults aged 66–76 years (Prokopidis et al., 2023). Despite these positive results, there are still limited health supplements that can robustly enhance memory function, with lack of research on their mechanisms. In this context, traditional Chinese medicine offers a rich repository of botanical candidates for promoting brain health and memory (Sun et al., 2013). *Cistanche tubulosa*, also known as “desert ginseng,” is widely used in traditional Chinese medicine and is believed to have effects such as kidney-yang tonification, laxative properties, fatigue relief, and anti-aging benefits (Zhou et al., 2023; Zhang S. et al., 2024; Zhang X. Y. et al., 2024). It is rich in metabolites like phenylethanol glycosides, iridoid ethers, lignans, polysaccharides, vitamins, and trace elements, all of which exhibit neuroprotective potential (Zhou et al., 2023). *Ginkgo biloba*, another ancient botanical drug, is well-known for its cognitive-enhancing properties (Morato et al., 2023). Its leaf extract, rich in flavonoids such as ginkgolides and bilobalide, is widely used in health foods and medicines for its antioxidant, anti-inflammatory, and neuroprotective effects (Noor et al., 2022; Li et al., 2023). Studies have shown that *Ginkgo biloba* can increase cerebral blood flow and improve memory and cognitive function (Mas et al., 2011).

While preliminary RCTs suggest both botanical drugs may improve cognition (Kan et al., 2021; Chen et al., 2024), their synergistic effects and impact on specific brain networks remain poorly characterized. To bridge this gap, the Nutrilite Health Institute developed a combined *Cistanche tubulosa*-*Ginkgo biloba* (CG) tablet. This randomized, double-blind, placebo-controlled trial was designed to evaluate CG’s efficacy in enhancing memory and elucidates its effects on brain structure and function through integrated neuropsychological testing and MRI. We specifically hypothesized that CG induced cortico-cerebellar reorganization, a potential mechanism for memory enhancement.

## 2 Materials and methods

### 2.1 Study design and study product

This study is a randomized, double-blind, placebo-controlled clinical trial, registered with the Chinese Clinical Trials Registry (ChiCTR2400084102). The trial was conducted from February 2019 to December 2020 at the PLA General Hospital, Beijing, China, and was in accordance with the Declaration of Helsinki standards and guidelines.

The study product is a health supplement developed by the Nutrilite Health Institute of Amway, containing a combination of extracts from *Cistanche tubulosa* (Schenk) Wight ex Hook.f. [Orobanchaceae] and *Ginkgo biloba* L. [Ginkgoaceae]. Each CG tablet contained 150 mg of *Cistanche* extract (standardized to ≥28.0% echinacoside, yielding ≥42 mg) and 60 mg of *Ginkgo biloba* extract (standardized to ≥25.0% total flavonol glycosides and ≥6.0% terpene lactones, yielding ≥15 mg and ≥3.6 mg, respectively). Functionally relevant metabolites comprised 2.85 g total flavonol glycosides and 7.55 g echinacoside per 100 g of tablets (Patent ID US9737582B2, US20150320818A1). More information and determination methods were detailed in Supplementary Table S1.

### 2.2 Participants

The inclusion criteria were as follows: (1) healthy subjects aged between 30 and 65 years, good overall health with no significant disorders of the brain, heart, liver, kidneys, lungs, or hematological system; (2) no history of long-term medication use; (3) no prior participation in similar tests (e.g., memory quotient or IQ tests); and (4) no use of drugs or health supplements related to memory improvement within the past year. All participants provided written informed consent. The exclusion criteria were: (1) pregnant or lactating women; (2) individuals allergic to health supplement ingredients; (3) patients with severe systemic diseases; (4) recent use of substances that could influence the study’s outcomes; (5) non-compliance with study protocol; or (6) incomplete baseline data that could impact the efficacy or safety evaluation. Subjects were balanced based on education level, age, and gender, and were subsequently randomized into either the CG intervention group or the placebo control group.

### 2.3 Intervention

In this double-blind trial, participants were randomized to receive either the CG tablets or sensorially indistinguishable placebo tablets. All participants were instructed to take two tablets daily (one tablet in the morning and one in the evening, approximately 12 h apart) for 30 days, adhering to the manufacturer’s recommended dosage. Placebo tablets contained glucose, microcrystalline cellulose, corn starch, caramel pigment, and inert excipients matched to CG tablets in appearance, taste, and texture. Participants consumed their assigned intervention for 30 days under strict double-blind conditions.

### 2.4 Clinical memory scale

Memory function was assessed at baseline and on Day 30 using an online clinical memory scale developed by the Psychology Institute of the Chinese Academy of Sciences. This scale aligns with the 2003 “Technical Standards for Testing and Assessment of Health Food” and is specifically designed to evaluate memory enhancement interventions (Inspection, 2003). The assessment was conducted at baseline and post-trial. The scale included the following subtests: (1) directional memory (DM): Participants were required to recall 12 target words from a list of 24, which included distractor items. This primarily assessed working memory and episodic memory under conditions of selective attention and interference control. Participants must actively maintain target words while inhibiting distractors, reflecting the ability to encode and retrieve specific items within a context. (2) associative learning (AL): Involved learning six pairs of words (three with logical connections and three without), tested in various sequences over three trials. This evaluated associative memory, a core component of episodic memory formation. It specifically tests the ability to bind different pieces of information (word pairs), which is fundamental for learning new facts and personal experiences. The inclusion of both logically related and unrelated pairs examined the efficiency of strategic versus rote associative encoding. (3) graphic free recall (GFR): Participants recalled 15 sequentially presented images of common daily items. This measured visual episodic memory and free recall ability. Recalling a series of common images tested the encoding, consolidation, and retrieval of visual information without cues, which was indicative of everyday memory for events and objects. (4) meaningless graphics recognition (MGR): Involved the recognition of five types of abstract graphic stimuli. This assessed visual recognition memory and pattern separation ability. Recognizing abstract graphics among distractors relied on the ability to form and retain distinct, non-verbal memory traces, tapping into perceptual representation memory systems. (5) portrait memory (PM): Participants memorized and later recalled details (name, occupation, preferences) associated with six portraits. This was a complex task that integrated episodic memory (binding multiple features—face, name, occupation, preference into a coherent episode) and semantic memory (recalling factual details). It closely simulated real-world social memory demands. The total score was calculated by summing the results from the five subtests, and the Memory Quotient (MQ) was derived from this total score (Luo et al., 2013; Zhang et al., 2017). All test administrators underwent training on the software system, and the assessments were conducted in a controlled, uninterrupted environment.

### 2.5 MRI

Resting-state functional MRI (fMRI) technology leverages the spontaneous fluctuations of blood oxygenation level-dependent (BOLD) signals to obtain functional brain imaging in a non-invasive and relatively fast manner. This technique measures hemodynamic changes induced by neuronal activity across the whole brain at multiple time points (Chu et al., 2021). Whole-brain MRI was conducted at the PLA General Hospital using a 3.0T Discovery MR 750 scanner (GE Healthcare, Milwaukee, WI, United States). Participants willing to undergo MRI testing were randomly selected, and both 3D-T1 high-resolution structural images and fMRI scans were performed at baseline and on Day 30.

The MRI data were processed using Statistical Parametric Mapping software (SPM12, http://www.fil.ion.ucl.ac.uk/spm) and the toolkit DPARSF (http://rfmri.org/DPARSF). Slice timing corrections were applied to all datasets. A 2x2 mixed analysis of variance (ANOVA) model, referred to as flexible ANOVA in SPM, was established to analyze the data. This model included both within-group and between-group factors. An F-test was used to determine the significance of main effects (group and time) and interaction effects. Post-hoc T-tests were then performed to further examine significant differences within and between groups. Areas with organic changes in gray matter volume were selected as seed regions, and known cognition-related regions were further selected as target region of interest (ROI). Further, the association of changes of gray matter volume or fractional amplitude of low-frequency fluctuations (fALFF) and clinical memory scale were tested to determine the potential causal association.

### 2.6 Statistical analysis

Descriptive statistics were reported as mean ± standard deviations (SD) for continuous variables and percentage for categorical variables. All primary analyses were based on the intention-to-treat (ITT) principle. Baseline categorical variables were analyzed using the Chi-square test or Fisher’s exact test, while continuous variables were assessed using the independent sample t-test. Changes within groups on the clinical memory scale were evaluated using the paired t-test and changes between groups were tested using independent t-test. In addition, to explore the influence of demographic factors on CG tablet efficacy, subgroup analyses were performed, stratified by age (30–49 vs. 50–65 years) and gender. For the MRI population, within-group changes and between-group differences were assessed using the Mann-Whitney U test due to the limited sample size. Associations between ROI and clinical memory scale score were tested using linear regression. A significance level of 0.05 was applied for all tests unless otherwise specified. Statistical analyses were conducted using R software (Version 4.2.2).

## 3 Results

### 3.1 Baseline characteristics of the study subjects

**FIGURE 1:**
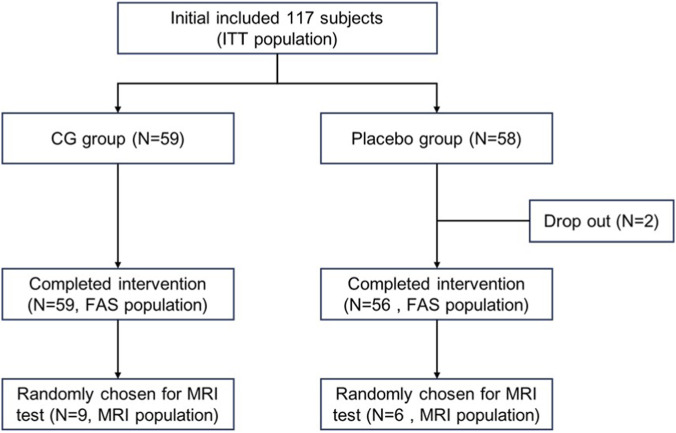
Study flow chart.

The study initially enrolled 117 participants (ITT population), with 59 randomized to the CG group and 58 to the placebo group (Figure 1). Demographic characteristics and baseline neuropsychological assessments are summarized in Table 1. The cohort had a mean age of 48.2 ± 6.8 years with 28.2% male representation. Two participants of the placebo group withdrew from the study due to personal reasons. A neuroimaging subsample (n = 15; CG = 9, placebo = 6) underwent serial MRI assessments at baseline and Day 30, with age ranging from 31 to 50 years. Importantly, baseline comparisons demonstrated no significant intergroup differences in age, gender distribution, educational attainment, or memory performance metrics across both the full cohort and MRI subsample (all p > 0.05), indicating successful randomization.

**TABLE 1:** Baseline Sociodemographic characteristics and clinical memory scale.

Indicator	ITT population			MRI population		
	CG group (N = 59)	Placebo (N = 58)	P-value	CG group (N = 9)	Placebo (N = 6)	P-value
Age	47.5 ± 10.0	48.8 ± 10.5	0.4943	40.3 ± 6.86	39.8 ± 6.01	0.8840
Gender	​	​	0.0565	​	​	0.9999
Male	12 (20.3%)	21 (36.2%)	​	2 (22.2%)	2 (33.3%)	​
Female	47 (79.7%)	37 (63.8%)	​	7 (77.8%)	4 (66.7%)	​
Education	​	​	0.0520	​	​	0.3455
High school or below	11 (18.6%)	9 (15.5%)	​	0 (0%)	0 (0%)	​
Bachelor	34 (57.6%)	23 (39.7%)	​	6 (66.7%)	3 (50.0%)	​
Master or above	14 (23.7%)	26 (44.8%)	​	3 (33.3%)	3 (50.0%)	​
Directional memory	21.3 ± 6.00	22.2 ± 5.28	0.3991	23.6 ± 6.04	24.3 ± 5.85	0.8080
Associative learning	32.5 ± 5.05	32.1 ± 5.40	0.7313	32.4 ± 5.10	32.5 ± 6.25	0.9859
Graphic free recall	25.7 ± 10.2	26.9 ± 9.03	0.5037	27.4 ± 10.3	24.7 ± 10.8	0.6289
Meaningless graphics recognition	21.1 ± 6.56	21.6 ± 6.12	0.6852	19.7 ± 5.92	20.2 ± 7.19	0.8906
Portrait memory	21.1 ± 6.70	22.1 ± 5.79	0.3735	23.0 ± 8.06	21.5 ± 3.51	0.6314

Data are presented as Mean ± SD, or Number (proportion). Nominal variables were analyzed using the Chi-square test or Fisher’s exact test, while continuous variables were assessed using the independent sample t-test in ITT population and Mann-Whitely U test in MRI population due to the limited sample size.

### 3.2 Neuropsychological outcomes

**FIGURE 2:**
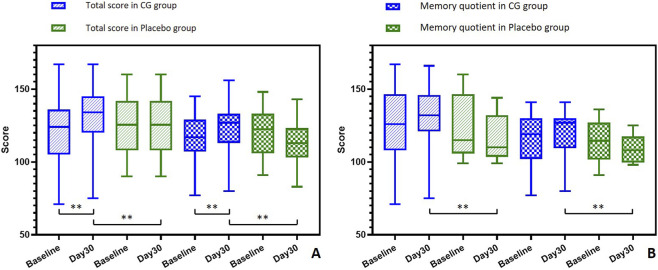
Total score of clinical memory scale and memory quotient at baseline and Day 30 in ITT population **(A)** and MRI population **(B)**. For the ITT population, changes within groups were evaluated using the paired t-test. Different between groups were evaluated using the independent t-test. For the MRI population, within-group changes and between-group differences were assessed using the Mann-Whitney U test due to the limited sample size. **: p < 0.01.

As shown in Table 2, the ITT analysis showed that participants in the CG group exhibited significant within-group improvements on DM, AL, GFR, and PM domains by Day 30 (all p < 0.01). Between-group comparisons revealed significantly greater improvement in the CG group compared to the placebo group for DM (mean change of 3.05 in the CG group vs. −1.21 in the placebo group, p = 0.0035), AL (1.86 vs. 0.16, p = 0.0015), and GFR (3.76 vs. −1.24, p = 0.0144). MGR scores decreased significantly within both groups post-intervention. However, the CG group showed a significantly smaller decline compared to the placebo group (−4.34 vs. −9.57, p = 0.0007). As shown in Figure 2, the CG group showed significant increase in the total score of clinical memory scale and MQ, and was significantly higher than those in the placebo group after intervention (P < 0.01).

**TABLE 2:** Clinical memory scale at Day 30 in both groups (Intention-to-Treat Analysis).

Indicator	ITT population				MRI population			
	CG group (N = 59)		Placebo (N = 58)		CG group (N = 9)		Placebo (N = 6)	
	Day 30	Change	Day 30	Change	Day 30	Change	Day 30	Change
Directional memory	24.3 ± 6.30	3.05 ± 6.30	21.1 ± 5.41	−1.21 ± 5.60	25.6 ± 6.62	2.00 ± 6.26	21.8 ± 5.00	−2.50 ± 4.18
*P*-value 1	**0.0005**		0.1062		0.3986		0.5196	
*P*-value 2	**0.0035**				0.1231			
Associative learning	34.3 ± 3.48	1.86 ± 4.27	32.3 ± 3.50	0.16 ± 5.03	34.8 ± 1.92	2.33 ± 4.53	32.5 ± 3.99	0.00 ± 5.80
*P*-value 1	**0.0014**		0.8151		0.6227		0.7475	
*P*-value 2	**0.0015**				0.2891			
Graphic free recall	29.4 ± 8.73	3.76 ± 6.79	25.6 ± 8.04	−1.24 ± 5.89	31.0 ± 10.5	3.56 ± 2.92	24.2 ± 8.01	−0.50 ± 4.51
*P*-value 1	**<0.0001**		0.1137		0.1422		0.9999	
*P*-value 2	**0.0144**				0.08635			
Meaningless graphics recognition	16.8 ± 7.19	−4.34 ± 6.94	12.0 ± 7.51	−9.57 ± 5.82	12.9 ± 9.75	−6.78 ± 5.91	8.50 ± 7.12	−11.7 ± 4.89
*P*-value 1	**<0.0001**		**<0.0001**		0.1435		**0.0220**	
*P*-value 2	**0.0007**				0.3370			
Portrait memory	25.2 ± 6.91	4.10 ± 7.54	23.0 ± 5.74	0.91 ± 6.23	24.1 ± 7.59	1.11 ± 7.99	25.3 ± 5.57	3.83 ± 5.42
*P*-value 1	**0.0001**		0.2685		0.6888		0.2257	
*P*-value 2	0.0650				0.8589			

Data are presents as Mean ± SD. P-value 1: changes within groups evaluated using the paired t-test. P-value 2: Difference between groups after intervention evaluated using independent t-test. For the MRI population, within-group changes and between-group differences were assessed using the Mann-Whitney U test due to the limited sample size. Numbers in bold indicate *P* < 0.05.

After diving the participants into two groups based on age, both age below 50 years and above 50 years groups showed significant increase in DM, GFR, PM, total score of clinical memory scale, and MQ after CG intervention (Table 3). Although the CG group in the subgroup with age over 50 showed significantly better outcomes in terms of DM, AL, GFR, and PM compared to the placebo group, this significant result was not observed in the subgroup with age under 50. This might be due to the fact that in the subgroup of patients under the age of 50, the baseline values of DM, AL, GFR and PM in the CG group were slightly lower than those in the placebo group. Furthermore, the total scores and MQ values of both subgroups after the intervention showed that the CG group was significantly better than the placebo group.

**TABLE 3:** Subgroup analysis of clinical memory scale at Day 30 in both groups based on age or gender (ITT analysis).

Subgroup	Age <50 years				Age ≥50 years			
Indicator	CG group (N = 34)		Placebo (N = 25)		CG group (N = 25)		Placebo (N = 33)	
	Baseline	Change	Baseline	Change	Baseline 30	Change	Baseline	Change
Directional memory	20.6 ± 5.81	3.59 ± 6.47	22.4 ± 4.75	−1.56 ± 6.63	22.3 ± 6.22	2.32 ± 6.11	22.2 ± 5.36	−0.94 ± 4.77
P-value 1	**0.0028**		0.2508		0.0696		0.2663	
P-value 2	0.0647				**0.0181**			
Associative learning	32.4 ± 4.43	1.38 ± 4.35	33.6 ± 4.49	−1.08 ± 3.90	32.5 ± 5.87	2.52 ± 4.16	31.1 ± 5.86	1.09 ± 5.61
P-value 1	0.0728		0.1794		**0.0058**		0.2727	
P-value 2	0.1398				**0.0021**			
Graphic free recall	24.6 ± 11.2	4.03 ± 6.34	27.3 ± 8.11	−1.48 ± 7.53	27.1 ± 8.65	3.40 ± 7.49	26.4 ± 9.65	−1.06 ± 4.37
P-value 1	**0.0008**		0.3354		**0.0325**		0.1731	
P-value 2	0.2548				**0.0067**			
Meaningless graphics recognition	20.8 ± 6.82	−4.76 ± 6.13	22.2 ± 7.08	−8.40 ± 5.60	21.2 ± 5.93	−3.76 ± 8.01	21.2 ± 5.51	−10.5 ± 5.91
P-value 1	**<0.0001**		**<0.0001**		**0.0275**		**<0.0001**	
P-value 2	0.2165				**0.0004**			
Portrait memory	20.8 ± 6.82	3.94 ± 8.11	22.4 ± 7.14	1.76 ± 7.80	21.5 ± 6.66	4.32 ± 6.85	21.8 ± 4.53	0.27 ± 4.75
P-value 1	**0.0078**		0.2701		**0.0043**		0.7434	
P-value 2	0.7728				**0.0242**			
Total score	119.5 ± 23.8	9.97 ± 12.9	128.0 ± 18.2	−9.04 ± 14.1	124.7 ± 17.8	10.4 ± 11.7	122.7 ± 20.1	−8.48 ± 11.6
P-value 1	**<0.0001**		**0.0037**		**0.0002**		**0.0002**	
P-value 2	**0.0397**				**<0.0001**			
Memory quotient	116.0 ± 17.1	7.62 ± 11.3	121.5 ± 14.1	−7.08 ± 11.1	117.2 ± 13.1	8.76 ± 8.92	119.3 ± 15.7	−6.97 ± 10.3
P-value 1	**0.0004**		**0.0038**		**<0.0001**		**0.0005**	
P-value 2	**0.0150**				**0.0005**			

Subgroup	Male				Female			
Indicator	CG group (N = 12)		Placebo (N = 21)		CG group (N = 47)		Placebo (N = 37)	
	Baseline	Change	Baseline	Change	Baseline 30	Change	Baseline	Change
Directional memory	22.8 ± 5.81	3.59 ± 3.50	24.0 ± 4.83	−3.05 ± 4.74	20.9 ± 6.17	2.91 ± 6.86	21.3 ± 4.99	−0.16 ± 5.84
P-value 1	**0.0046**		**0.0080**		**0.0055**		0.8668	
P-value 2	**0.0104**				**0.0488**			
Associative learning	34.2 ± 3.54	−0.25 ± 3.05	32.8 ± 5.28	−0.19 ± 5.03	32.0 ± 5.31	2.40 ± 4.40	31.8 ± 5.52	0.35 ± 5.09
P-value 1	0.7816		0.8639		**0.0005**		0.6770	
P-value 2	0.2398				**0.0027**			
Graphic free recall	27.0 ± 9.67	3.33 ± 4.16	27.3 ± 8.03	−1.19 ± 5.87	25.3 ± 10.4	3.87 ± 7.35	26.5 ± 9.54	−1.27 ± 5.98
P-value 1	**0.0181**		0.3638		**0.0007**		0.2042	
P-value 2	0.1222				**0.0451**			
Meaningless graphics recognition	21.9 ± 7.42	−6.33 ± 9.62	21.6 ± 6.99	−9.24 ± 5.98	20.9 ± 6.39	−3.83 ± 6.11	21.6 ± 5.71	−9.76 ± 5.79
P-value 1	**0.0436**		**<0.0001**		**<0.0001**		**<0.0001**	
P-value 2	0.3789				**0.0006**			
Portrait memory	23.3 ± 7.24	4.58 ± 8.03	23.1 ± 5.97	0.52 ± 6.18	20.5 ± 6.51	3.98 ± 7.50	21.5 ± 5.63	1.14 ± 6.33
P-value 1	0.0736		0.7017		**0.0007**		0.2826	
P-value 2	**0.0476**				0.1792			
Total score	129.2 ± 21.7	6.00 ± 11.3	128.9 ± 19.0	−10.4 ± 11.1	119.8 ± 21.2	11.2 ± 12.5	122.8 ± 19.3	−7.78 ± 13.4
P-value 1	0.0931		**0.0004**		**<0.0001**		**0.0012**	
P-value 2	**0.0209**				**0.0004**			
Memory quotient	120.2 ± 13.8	5.17 ± 8.96	122.3 ± 14.2	−8.24 ± 8.60	115.6 ± 15.8	8.85 ± 10.6	119.1 ± 15.4	−6.32 ± 11.5
P-value 1	0.0712		**0.0003**		**<0.0001**		**0.0019**	
P-value 2	**0.0260**				**0.0008**			

Data are presents as Mean ± SD. P-value 1: changes within groups evaluated using the paired t-test. P-value 2: Difference between groups after intervention evaluated using independent t-test. Numbers in bold indicate *P* < 0.05.

After stratifying the participants by gender, females demonstrated a significant increase in DM, AL, GFR, PM, the total clinical memory score, and MQ after the CG intervention (Table 3). A similar trend was observed in males, although only the improvements in DM and GFR reached statistical significance, a finding potentially attributable to the smaller sample size of males within the CG group.

Analysis of the MRI population (n = 15) showed a significant within-group decline in MGR for the placebo group (p = 0.047), but no significant changes in other domains were detected, likely attributable to limited sample size. In the full cohort, global cognitive metrics including the total composite score and MQ showed significant between-group improvement favoring the CG group (p < 0.001). As depicted in Figure 2, these improvements in total score and MQ favoring the CG group were also evident in the neuroimaging subsample, although statistical significance was not reached due to the small sample size. However, significant higher total score and MQ in the CG group than the placebo group after intervention can be found.

### 3.3 Neuroanatomical alterations

**FIGURE 3:**
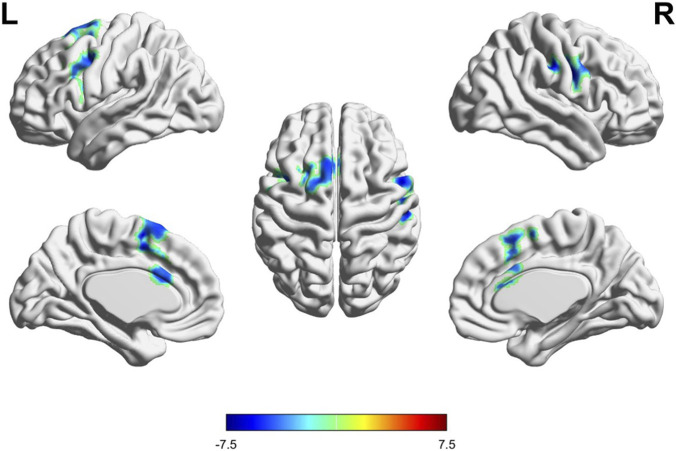
Changes in gray matter volume (GMV) post-intervention in the CG group. Brain renderings depict regions with significant increases (yellow/red) and decreases (blue/cyan) in GMV following the intervention in the CG group (p < 0.05, FDR corrected). Different perspectives including left, right, top, and medial views are presented.

Voxel-based morphometry revealed significant GMV reductions localized to three motor-cognitive hubs exclusively within the CG group (FWE-corrected cluster-level p < 0.05; Table 4; Figure 3): 1. Left precentral gyrus (Brodmann area 4, peak MNI: −40, −16, 22), extending to the adjacent middle frontal gyrus. 2. Right precentral gyrus (Brodmann area 4, peak MNI: 54, 4, 34), with partial overlap in the inferior frontal gyrus. 3. Left supplementary motor area (SMA, peak MNI: −8, 4, 58), involving the medial frontal cortex. No significant GMV changes were observed in the placebo group at the same statistical method, confirming the intervention-specific nature of these structural alterations.

**TABLE 4:** Brain regions with significant different values of gray matter volume (GMV) and Low-Frequency Fluctuations (fALFF) in the CG groups before and after intervention.

Indices	Clusters	MNI coordinates			Brain regions	Voxels	T values	*P*-value 1	*P*-value 2
		X	Y	Z				
GMV	Cluster 1	−40	16	22	Precental gyrus (L)	559	−6.1466	0.007	<0.001
	Cluster 2	−8	4	58	Supplementary motor area (L)	1997	−6.4134	<0.001	<0.001
	Cluster 3	54	4	34	Precental gyrus (R)	719	−6.9848	0.005	<0.001
fALFF	Cluster 1	3	−72	−6	Cerebelum 6 (L)	127	8.4349	0.019	<0.001
		12	−78	−12	Cerebelum 6 (L)	127	7.8301	0.026	<0.001

GMV, gray matter volume; ALFF, amplitude of low frequency fluctuation; MNI, montreal neurological institute; L, left, R, right. P-value 1, main effect between groups using F test. P-value 2, difference between groups using T-test.

### 3.4 Functional brain changes

**FIGURE 4:**
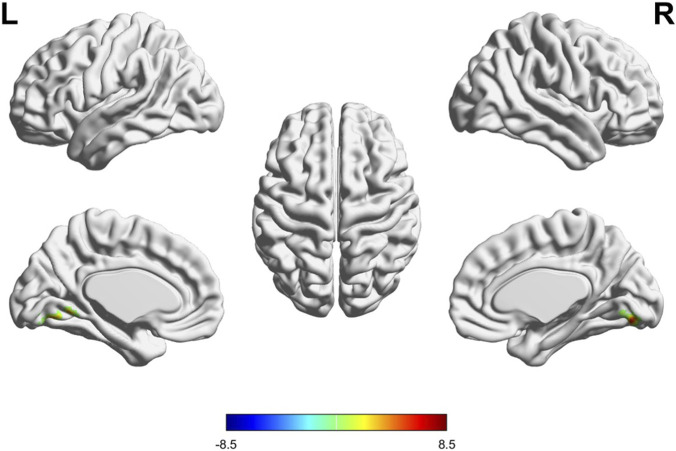
Changes of fractional Amplitude of Low-Frequency Fluctuations (fALFF) across different brain regions post-intervention in the CG group. Brain renderings illustrate regions with significant increases (yellow/red) and decreases (blue/cyan) in fALFF following the intervention in the CG group (p < 0.05, FDR corrected). Different perspectives including left, right, top, and medial views are presented.

Resting-state fMRI analysis of fALFF demonstrated significant CG-specific increases in fALFF within the left cerebellar lobule VI (peak MNI coordinates: 3, −72, −6; 12, −78, −12) - a key node in cognitive-motor networks (Stoodley and Schmahmann, 2009) (Figure 4). Post-hoc analysis confirmed robust fALFF increases in the CG group (e.g., peak T = 8.43, p < 0.001; peak T = 7.83, p < 0.001). In contrast, no significant fALFF changes were observed in the placebo group across cerebellar-cortical circuits.

### 3.5 Correlations between changes of memory scale and functional magnetic resonance imaging metrics

**FIGURE 5:**
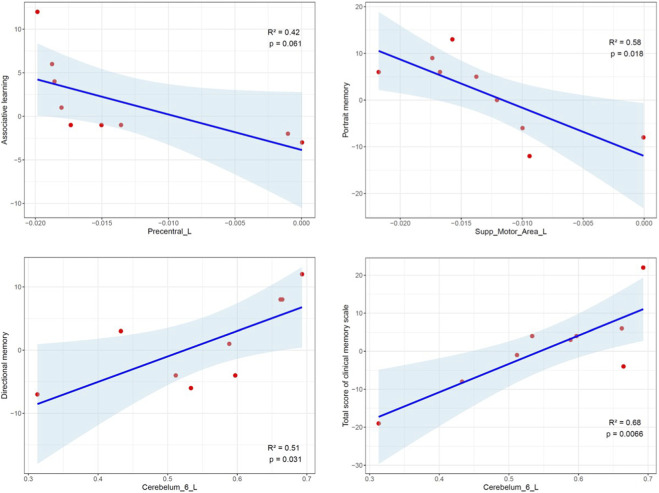
Significant correlation between changes of GMV or fALFF and clinical memory scale The red scattered points represent the observed values of the MRI population, while the blue solid line and the shaded area represent the fitted linear regression and 95% confidence interval.

Within the CG group, exploratory correlation analyses were conducted between changes in clinical memory scale scores and changes in neuroimaging metrics (Figure 5). 1. GMV Changes: A significant negative correlation was observed between the change in PM score and the change in GMV within the left SMA (R^2^ = 0.58, p = 0.018). This indicates that greater improvement in PM was associated with greater reduction in left SMA GMV. Similarly, a negative correlation between the change in AL score and the change in GMV within the left precentral gyrus approached statistical significance (R^2^ = 0.42, p = 0.061), suggesting a potential association where greater AL improvement tended to accompany greater left precentral GMV reduction. 2. fALFF Changes: Significant positive correlations were found between the change in fALFF within left cerebellar lobule VI and both the change in DM score (R^2^ = 0.51, p = 0.031) and the change in total memory score (R^2^ = 0.68, p = 0.007). These findings demonstrate that increased spontaneous neural activity in the cerebellum is robustly linked to enhanced memory performance following CG intervention.

### 3.6 Safety profile

In the present 30-day trial, the intervention was well-tolerated by all participants. No adverse events (AEs) were spontaneously reported by any participant in either the intervention or control group throughout the study period. No participants withdrew from the study due to intervention-related discomfort.

## 4 Discussion

This 30-day randomized controlled trial provides novel evidence that daily CG supplementation enhances memory performance in healthy adults through distinct neuroplastic mechanisms. Our multimodal findings demonstrate that CG intervention not only improved objective neuropsychological metrics but also induced concurrent structural and functional reorganization within cognitive-motor networks. This was achieved alongside a favorable safety profile, with no treatment-related adverse events reported.

The results demonstrated that CG tablets significantly improved cognitive performance, as evidenced by increased scores in the DM, AL, GFR, and PM subtests of the Clinical Memory Scale. Moreover, the intervention led to a significant increase in the total memory score and MQ compared to the placebo group. The DM and AL subtests, which primarily assess learning abilities associated with left-hemispheric functions, and the GFR subtest, which discriminates between memory types with minimal influence from literacy levels (Zhang et al., 2017), both showed remarkable improvement. The PM subtest, specifically sensitive to bilateral hippocampal integrity and facial-name recognition (Zhang et al., 2017), also showed significant enhancement. These findings were consistent with previous studies reporting the cognitive-enhancing effects of *Cistanche tubulosa*, which has been shown to improve learning and memory in APP/PS1 mice (Wang et al., 2022) and enhance various cognitive functions in older adults (Chen et al., 2024). Similarly, *Ginkgo biloba* has been found to enhance spatial working memory, recognition memory, and both short- and long-term recall in naïve rats (Tomova et al., 2021), though its effects in human populations have been less pronounced (Liu et al., 2019). Conversely, the observed reduction in MGR scores across both groups suggests this non-verbal, right-hemisphere-associated test may be susceptible to practice interference, the inherent difficulty of meaningless graphical stimuli, or potential stochastic variations in the computer-generated task order (Zhang et al., 2017).

Regarding fMRI results, clusters of reduced GMV were identified post-intervention in the precentral gyrus and SMA, regions traditionally associated with motor control (Lee et al., 2022). The precentral gyrus was primarily considered to be the primary motor cortex, responsible for controlling voluntary movement on the opposite side of the body. Additionally, the left precentral gyrus was also involved in semantic analysis and memory-related language processing. In a working memory task, the activation of the left anterior central gyrus was positively correlated with the average reaction time (Gabrieli et al., 1998). According to the Brodmann partition, the left anterior central gyrus corresponds to BA4, which may also contain a part responsible for motor sequence planning, working memory, plot, long-term memory and semantics BA6 region (Gabrieli et al., 1998; Miotto et al., 2014). Studies have shown that the left SMA is integral to working memory, particularly regarding executive functions and sequential processing across action, language, and music domains (Tanaka and Kirino, 2017). The observed reduction in GMV might be related to increased efficiency or plasticity and the enhancement of synaptic pruning, a process that improved the efficiency of neural signaling by eliminating redundant neural connections and preserving efficient pathways (Wang et al., 2015). For example, a decline in GMV in the SMA was observed after cognitive behavioral therapy, which was thought to be likely related to enhanced emotional regulation and cognitive control (Qiu et al., 2019). In our study, the significant negative correlations between PM/AL improvements and localized GMV reductions further suggest that neural remodeling is a complex process where “larger” does not inherently equate to “better.” CG intervention might facilitate a transition toward a more streamlined cortical structure while optimizing functional connectivity with other brain regions (West et al., 2024).

In addition, fALFF in left cerebellum was significantly upregulated by CG intervention, suggesting a compensatory mechanism wherein the brain optimizes its functional networks to maintain or enhance cognitive performance despite structural changes (Stern et al., 2019; Jha and Jha, 2023). Increased activity in this region has been associated with improved visuospatial processing and memory consolidation (Zhang et al., 2023; Vias and Dick, 2017), which aligns with the cognitive improvements noted in this study. The reduction in GMV may reflect neuroplastic changes, as the brain undergoes structural remodeling in response to the cognitive demands imposed by the intervention (Zotey et al., 2023). Such changes are not rare in studies involving cognitive training or pharmacological interventions aimed at enhancing cognitive function (Engvig et al., 2010; Takeuchi et al., 2011). While these findings should be interpreted with caution due to the inherent nuances of neuroimaging metrics, they suggest that CG tablets promote broader cognitive resilience through enhanced neural efficiency and functional reorganization.

The memory and cognitive-enhancing effects of the combined extract of CG were hypothesized to arise from a multi-targeted synergy. *Ginkgo biloba* extract (GBE), standardized to flavonoid glycosides and terpene lactones (e.g., ginkgolides), primarily modulated cerebral vascular function and redox homeostasis (Singh et al., 2019). Its constituents acted as potent platelet-activating factor (PAF) receptor antagonists, reducing blood viscosity and enhancing microcirculation, thereby improving cerebral blood flow and the delivery of oxygen and glucose to metabolically active neurons—a fundamental requirement for sustaining long-term potentiation (LTP) (Chen et al., 2018). Complementarily, GBE exerted direct antioxidant activity by scavenging free radicals, mitigating oxidative stress that contributed to neuronal aging (Singh et al., 2019). In parallel, *Cistanche tubulosa* extract (CTE), rich in echinacoside and acteoside, targeted neuronal integrity and plasticity. CTE upregulated neurotrophic factors such as brain-derived neurotrophic factor (BDNF) and nerve growth factor (NGF), promoting neurite outgrowth and synaptic stabilization (Li et al., 2016). Additionally, CTE inhibited microglial overactivation, reducing the release of pro-inflammatory cytokines (e.g., TNF-α, IL-1β), which, together with the antioxidant capacity of GBE, provided a layered defense against neuroinflammation and oxidative damage (Liao et al., 2023). Collectively, while GBE optimized the cerebral hemodynamic milieu, CTE directly bolstered synaptic resilience and neurotrophic support, yielding a multifaceted intervention that addressed the complex etiology of cognitive decline more comprehensively than monotherapies.

From a clinical perspective, the observed improvements in memory-related subtests imply that CG tablets could be beneficial in enhancing short-term memory, associative learning, visuospatial processing, and facial recognition—domains critical for daily autonomy and complex problem-solving (Swanson and Kim, 2007). The clinical significance lies in the potential application of CG tablets as a non-invasive, easily administered intervention for improving cognitive function in various populations. For example, older adults experiencing age-related cognitive decline or individuals with mild cognitive impairment (MCI) might benefit from such an intervention. Beyond memory, the observed improvements may also reflect enhanced attention, executive function, and learning capacity, which are integral to complex cognitive processes (McCabe et al., 2010), making CG tablets a holistic candidate for cognitive maintenance in both healthy and clinical populations.

This study combined apparent scale results with MRI screening to validate the potential benefits of CG extracts on memory and cognitive function. However, several limitations should be noted. First, the sample size for the MRI subset was limited, which may have contributed to the lack of significant findings on the Clinical Memory Scale in this population. Similarly, in the subgroup analysis, the limited sample size also led to some statistically insignificant results. Second, the short 30-day intervention period necessitates future longitudinal studies to establish long-term efficacy. Lastly, further molecular investigations are required to definitively map the underlying signaling pathways through which CG modulates the central nervous system (Jha et al., 2022).

## 5 Conclusion

In conclusion, this RCT demonstrated the efficacy of CG tablets in enhancing memory function and supported their potential as a clinical intervention for cognitive health. Neuroimaging revealed that CG supplementation modulated neural plasticity through cortico-cerebellar reorganization, evidenced by increased fALFF in the cerebellar culmen and structural adaptations in cortical regions. These findings suggested CG tablets might as a proactive strategy to maintain cognitive vitality in middle-aged and elderly populations at risk of age-related decline. Future longitudinal studies are warranted to assess long-term efficacy and expand these findings to broader clinical cohorts, such as individuals with MCI, to fully establish the therapeutic utility of CG in neurodegenerative prevention.

## Data Availability

The raw data supporting the conclusions of this article will be made available by the authors, without undue reservation.
