# Supplementary material for: Cistanche tubulosa-Ginkgo biloba combination enhances memory via cortico-cerebellar reorganization: a randomized controlled trial
Source: Front Pharmacol. 2026 Mar 2;17:1654013. doi: 10.3389/fphar.2026.1654013 (PMC12989481; doi:10.3389/fphar.2026.1654013)
Supplement: Supplementary file 1 [file Table1.docx]

Supplementary Material

**Supplementary Table 1 Details of the CG tablet**

| Raw material | Extracts from *Cistanche tubulosa* (Schenk) Wight ex Hook.f. [Orobanchaceae], extracts from *Ginkgo biloba* L. [Ginkgoaceae] |
| --- | --- |
| Ingredients | The formulation consisted of microcrystalline cellulose powder (containing microcrystalline cellulose and silicon dioxide), glucose (blended with maltodextrin), corn starch, croscarmellose sodium, silicon dioxide, and magnesium stearate. The tablets were finished with a coating system comprising hydroxypropyl methylcellulose (HPMC), glycerol, and carnauba wax. |
| Biomarker components | - echinacoside ≥7.55 g / 100 g - total flavonol glycosides ≥2.85 g / 100 g |
| Determination methods | - echinacoside: Samples containing echinacoside were dissolved in methanol, and the identification and quantification of echinacoside were performed using high-performance liquid chromatography (HPLC). - total flavonol glycosides: according to the method described for 'Ginkgo Leaf Tablets' under the 'Content Determination' section of the Pharmacopoeia of the People's Republic of China. |
| Extraction method | - *Cistanche tubulosa*   *Cistanche* was prepared through a series of processes, including rinsing, moistening, and pulverization, followed by aqueous extraction (three boiling cycles of 2 h, 1 h, and 1 h with 8, 6, and 6 volumes of water, respectively). The resulting extract was concentrated, subjected to ethanol precipitation, and purified via macroporous resin chromatography. The 40% ethanol eluate was collected, concentrated, and spray-dried (inlet temperature: 150~200°C; outlet temperature: 75~110°C) before final packaging.   - *Ginkgo biloba*   *Ginkgo* leaves were processed through pulverization followed by extraction (circulating extraction with 65% ethanol at 60~70°C for 3.5 h). The extract was concentrated, purified by resin adsorption, and re-concentrated. The final product was obtained by spray drying (inlet temperature: 195± 5°C; outlet temperature: 90 ± 5°C), followed by pulverization, sieving, and packaging. |

Patent ID US9737582B2, US20150320818A1.
